# Antibody-mediated clearance of tau in primary mouse microglial cultures requires Fcγ-receptor binding and functional lysosomes

**DOI:** 10.1038/s41598-019-41105-4

**Published:** 2019-03-15

**Authors:** Christian Rungsted Andersson, Jeppe Falsig, Jeffrey B. Stavenhagen, Søren Christensen, Fredrik Kartberg, Nina Rosenqvist, Bente Finsen, Jan Torleif Pedersen

**Affiliations:** 1 Department of Neurodegeneration, H. Lundbeck A/S, Copenhagen, Denmark; 2 Neurobiology Research, Institute of Molecular Medicine, University of Southern Denmark, Odense, Denmark; 3 Biology, Therachon, Basel, Switzerland; 4 Biologics, H. Lundbeck A/S, Copenhagen, Denmark

## Abstract

Neurodegenerative diseases such as Alzheimer’s disease are characterized by the progressive spreading and accumulation of hyper-phosphorylated tau protein in the brain. Anti-tau antibodies have been shown to reduce tau pathology in *in vivo* models and antibody-mediated clearance of tau exerted by microglia has been proposed as a contributing factor. By subjecting primary microglia cultured *in vitro* to anti-phospho-tau antibodies in complex with pathological tau, we show that microglia internalise and degrade tau in a manner that is dependent on FcγR interaction and functional lysosomes. It has recently been discussed if anti-tau antibody effector-functions are required for induction of tau clearance. Using antibodies with compromised FcγR binding and non-compromised control antibodies we show that antibody effector functions are required for induction of microglial clearance of tau. Understanding the inflammatory consequences of targeting microglia using therapeutic antibodies is important when developing these molecules for clinical use. Using RNA sequencing, we show that treatment with anti-tau antibodies increases transcription of mRNA encoding pro-inflammatory markers, but that the mRNA expression profile of antibody-treated cells differ from the profile of LPS activated microglia. We further demonstrate that microglia activation alone is not sufficient to induce significant tau clearance.

## Introduction

Tauopathies such as Alzheimer’s disease, frontotemporal dementia and parkinsonism linked to chromosome 17 (FTDP-17), and progressive supra-nuclear palsy (PSP) are diseases characterised by the intra-neuronal spreading of pathological forms of the microtubule associated protein tau through anatomically connected areas of the brain^1–3^.

The physiological role of tau is to stabilise the dynamic microtubule scaffold which requires frequent re-organization as part of a range of cellular homeostatic processes such as axonal transport and maintenance of cellular morphology as well as during neuronal development and synaptogenesis^4–8^. This requires interactions with microtubules dynamically regulated, in part, by phosphorylation of tau at several identified phosphorylation sites in the protein reducing its affinity for microtubules^2,9,10^. In experimental models of tauopathy, tau has been shown to seed pathology causing it to aggregate in the cytoplasm and to spread to postsynaptic neurons in a prion-like manner^11^. Hyperphosphorylated aggregated tau accumulates as neurofibrillary tangles (NFT) that eventually kills the affected neurons.

As NFT pathology correlates with the cognitive decline observed during Alzheimers disease^12^, the aggregation of tau is considered an important step in the neurodegenerative process, suggesting that it may be an interesting therapeutic target. Antibodies targeting tau have been reported to reduce tau pathology and prevent functional impairment in rodent models of tauopathies^13–17^. The mechanism of action of antibodies in the brain is not fully understood, and several immunotherapies targeting tau are currently in development^18^.

Microglia have been shown to internalise tau protein both *in vitro* and *in vivo*^19^ and it has been shown that therapeutic antibodies can potentiate uptake of tau into BV2 cells^20^ and induce microglial removal of tau from culture medium^21^. Microglia express pattern-recognition receptors (PRRs), toll-like receptors (TLRs) and scavenger receptor A (SRA) which mediate phagocytosis of non-antibody-bound targets. Microglia also express all of the four IgG antibody receptors FcγRI, IIb, III and IV^22^ which are involved in the receptor-mediated antibody-dependent internalisation of immune complexes destined for intracellular antibody-mediated degradation (IAMD). Internalisation of immune complexes by microglia happens through binding of antibody Fc domains protruding from the immune complexes to FcγRs on the cell surface^23^. Binding causes FcγRs to cluster on the cell surface, leading to phosphorylation of cytoplasmic motifs of the FcγRs^24^. Murine FcγRs can be categorised into either activating (FcγRI, III, and IV) or inhibitory (FcγRIIb) FcγRs that have either Immunoreceptor Tyrosine Activating Motifs (ITAM) or Immunoreceptor Tyrosine Inhibitory Motifs (ITIM), respectively. Binding of antibodies to activating FcγRs results in phosphorylation of ITAMs and down-stream activation pathways activating the cell, whereas binding to inhibitory FcγRIIb results in phosphorylation of ITIMs initiating inhibitory signalling pathways^24^. FcγR-dependent increases in the expression of microglial and macrophage markers of activation has been shown following *in vivo* formation of immune complexes in the brain^25^. Activation of microglia has also been shown to result in production and secretion of pro-inflammatory cytokines such as TNF and IL1b as well as production of reactive oxygen species and nitric oxide^26,27^. Although these markers are associated with microglial activation, this term should be used with caution as the microglia activation is not an all/nothing response, but rather covers a spectrum of activation patterns^27,28^.

The work presented in this study is carried out using primary mouse microglia treated with human pathological hyper-phosphorylated sarkosyl-insoluble P3 tau material purified from aged rTg4510 mice over-expressing human 0N4R P301L tau. We expose primary mouse microglia to P3 tau-antibody immune complexes and use these to study the internalisation and clearance of tau. Using two different approaches we show that FcγR interaction is necessary in order to trigger IAMD by microglia *in vitro*. We furthermore show that lysosomal function is required for microglial clearance of tau. To characterise the inflammatory profile of these cells following antibody treatment, we use RNA-sequencing (RNA-Seq) to describe the inflammatory status of these cells following antibody treatment.

## Methods

### Primary mouse microglial cultures

Cultures were prepared from post-natal NMRI BomTac mouse pups (Taconic) at 1–2 days of age. Whole brains were trypsin digested and made into a cell suspension. Cells were seeded in flasks pre-coated with 0.1 mg/ml poly-L-lysine (Sigma P1399) and maintained in DMEM supplemented with 10% heat-inactivated FCS and 1% Pen-Strep (Gibco 15140122). From DIV05-08 cultures received medium supplemented with 10 ng/ml Macrophage Colony Stimulating factor (M-CSF) (ThermoFisher RP-8615) diluted in PBS supplemented with 0.1% sterile filtered BSA (Sigma BSAV-RO). Following M-CSF treatment, microglia were isolated by orbital shaking at 150 RPM for 1 hour in an incubator and the supernatant was seeded in 96-well plates with 20,000 cells per well. Experiments were performed on the subsequent day.

### Purification of hyper-phosphorylated P3 tau from rTg4510 P301L mice

P3 tau was prepared from ten month old rTg4510 male and female mice expressing human P301L 0N4R tau by a method adapted from Sahara *et al*.^29^ without protease and phosphatase inhibitors. All animals used for preparation of hyper-phosphorylated P3 tau material were bred and maintained as described by Helboe *et al*.^30^. All animal experiments were performed in accordance with the European Communities Council Directive #86/609, the directives of the Danish National Committee on Animal Research Ethics, and Danish legislation on experimental animals (license no. 2014-15-0201-00339). In brief, whole brains excluding cerebella were individually homogenized in 10% w/v Tris-buffered saline [50 mMTris/HCl (pH 7.4), 274 mM NaCl, 5 mM KCl in ddH_2_O], ultra-centrifuged at 27,000 × g at 4 °C for 20 minutes. The supernatant (S1) was removed and the pellet (P1) was re-suspended to 20% w/v in buffer [10 mM Tris, (pH 7.4), 0.8 M NaCl, 10% Sucrose in ddH_2_O], homogenized and centrifuged at 27,000 × g for 20 minutes. The resulting S2 supernatant was incubated with 1% sarkosyl at 37 °C for 1 hour and then centrifuged at 150,000 × g at 4 °C for 1 hour. The resulting P3 pellet, containing the aggregated sarkosyl-insoluble tau, was resuspended in a TRIS/EDTA buffer. P3 fractions from the individual animals were verified to contain 64 kDa hyper-phosphorylated tau band by western blotting. Following validation, fractions were pooled into one batch which was used for subsequent experiments. Western blot was performed with rabbit anti-tau (Dako A0024) and mouse anti-β-actin (Sigma A5441) antibodies followed by goat anti-rabbit (LiCor 926-32211) and anti-mouse (Life Technologies A-21059) detection antibodies. A recombinant human tau protein ladder (Sigma-Aldrich T7951) containing all 6 tau isoforms was included for comparison. Scanning of membranes was performed using an Odyssey CLx scanner (Li-Cor).

### Tau internalisation and degradation assay

P3 tau was added to microglia at a concentration of 6.0 ± 1.1(SD) ng/ml as determined by total human tau ELISA (Thermofisher KHB0041). P3 tau was measured using treatment with 8 M urea for 30 minutes for monomerisation of aggregated tau prior to ELISA performed as decribed in the kit manual. Following addition, microglia were fixed at different time points using 4% PFA. Before fixation, treatment medium was removed and cells washed two times in PBS to remove extracellular tau. Unless otherwise specified, experimental treatments were always performed in medium without serum. In experiments where tau antibodies were included, P3 tau and antibodies were mixed in medium and pre-incubated at 37 °C for 30–45 minutes to allow for immune complex formation prior to addition to cells. Mixing was performed two times during incubation by repeated manual pipetting. In experiments using 10 µg/ml antibody the stochiometric ratio between antibody and P3 tau was 668:1. All experiments were performed in biological triplicates with n = 3 wells per treatment, n = 9 in total, unless otherwise specified.

### Antibodies used in antibody-degradation experiments

Anti-tau antibodies C10.2 and C10.2_D265A_ targeting pS396 tau were developed and manufactured as described in Fuller *et al*.^31^. Commercially available Tau5 (Thermofisher MS-247-PABX) targeting total tau and AT8 targeting tau phosphorylated at S202 and T205 (ThermoFisher MN1020) without azide were included for comparison. All included antibodies were IgG1 isotype.

### Fc-receptor blocking

Experiments were performed by pre-incubating microglia with the anti-mouse CD16/CD32 antibody Fc Block (BD Pharmingen) and control treatments diluted in medium with serum for 1.5 hours. Following pre-incubation, the anti-tau antibody: P3 tau immune complexes were added to cells in combination with specified concentrations of Fc Block in medium without serum. Experiments were performed in biological triplicates with n = 3 wells per treatment, n = 9 in total.

### Mouse FcγR binding assay

Antibody affinities for mouse FcγRs were determined using a custom assay developed by Cisbio. The assay relies on HEK293 cells expressing mouse FcγRs co-expressed with the functionally relevant gamma chain labeled with a terbium donor dye. Unlabeled antibodies compete with an acceptor labeled mouse IgG (IgG-d2) for binding to the receptor. When the dyes are in close proximity, the excitation of the donor with a laser triggers a Fluorescence Resonance Energy Transfer (FRET) towards the acceptor, which in turn fluoresces. Emission was measured at 620 nm (FRET donor) and 665 nm (FRET emission) and the ratio: 665 nm/620 nm × 10.000 was calculated. The calcualted ratios were converted to percentages of the maximum binding signal from the IgG-d2 conjugate. The unlabeled antibody present in the sample, competes for binding on the relevant receptor with the IgG-d2 conjugate and thereby prevents FRET from occurring. The specific signal modulates negatively and can be used to determine the relative IgG affinity for the FC-gamma receptor. Controls were: 1. cells without conjugate (only Tb) – negative control/FRET donor signal; 2. cells with conjugate (Tb + d2) and no competing IgG – 100% FRET signal and 3. buffer only – buffer control. All mouse assays were run as a fee-for-service at Cisbio.

### Immunocytochemistry and staining

Following fixation, cells were washed in KPBS [152.3 mM NaCl, 2.7 mM KCl, 1.9 mM NaH_2_PO_4_·H_2_O, 8.5 mM Na_2_HPO_4_ in distilled H_2_O] and then blocked and stained in KPBS supplemented with 0.5% w/v bovine serum albumin, 0.1% TritonX, and 5%vol normal swine serum (Jackson ImmunoResearch 014-000-121 diluted in 10 ml dd water). Tau detection was performed using a polyclonal rabbit anti-human-tau specific antibody (E1) targeting aa19-33 in the N-terminal of tau^32^ followed by anti-rabbit antibodies (Alexa A21206) and 1 µg/ml Hoechst 33342. For visualisation of microglia morphology wheat germ agglutinin (WGA) was used. WGA cells were stained live by incubation with 5 µg/ml tetramethylrhodamine-labeled WGA in HBSS (Invitrogen) for 10 minutes at room temperature. After WGA stain, cells were washed, fixed, permeabilized and stained as described above. Quantification of tau staining was performed using a Cellomics Arrayscan VTI setup (Thermofisher) and the accompanying software and algorithm. By assessment of nuclear morphology and staining intensity the algorithm excludes dead cells from analysis, and quantifies the area of fluorescent spots within a defined distance from the edge of the nuclear staining. The area of spots with staining intensities greater than a pre-defined background level is measured. Untreated controls, and cells treated with P3 alone were used as negative and positive controls, respectively, to calibrate the algorithm. All experimental treatments were performed with n = 3 wells per treatment and typically a total of 2400–3600 cells were measured per treatment. To ensure that data are not affected by differences in cell numbers the algorithm normalises the total spot area quantified to the number of cells included in analysis. For overview figure, please refer to Supplementary Fig. 2 in the Supplementary Dataset File.

### pHrodo labeling and antibody uptake experiments

Was performed using pHrodo™ Red, succinimidyl ester (Invitrogen P36600) according to the manufacturer manual using 10X molar excess of pHrodo and using 50 mM sodium borate buffer (pH 8.5) (Sigma-Aldrich) for labeling. Antibody protein concentrations were determined using Pierce™ BCA Protein Assay Kit (Thermo Scientific 23225) using a mouse IgG standard. All antibodies were diluted to 1 mg/ml and stained for 60 minutes with shaking in the dark before glycine (Sigma-Aldrich) was added to a final concentration of 150 mM to capture unbound pHrodo. Samples were then dialysed using microdialysis casettes with a 3.5 kDa molecular cut-off allowing glycine to leave the cassette, while retaining antibodies (Thermo Scientific 66333). Casettes were placed in 1 liter of sterile PBS with stirring for 3 hours at 4 °C. This was repeated two times with fresh PBS, before PBS was changed and dialysis was performed over night. A PBS control without antibody was included in the entire labeling procedure to control that no residual pHrodo would be present in the antibody solvent after labeling and dialysis. Antibody uptake experiments were performed as stated under description of the tau internalisation and degradation assay.

### Pharmacological inhibition of degradation compartments

Was performed by pre-incubating cells with inhibitors for 1 hour prior to addition of tau-antibody complexes. Pre-incubation was performed in medium supplemented with serum, subsequent treatments with immune complexes were performed in medium without serum. The following inhibitors were used: Bafilomycin A1 (Sigma B1793), Bortezomib (Cell Signalling Technology 2204S), Chloroquine (Sigma C6628), Epoxomicin (R&D Systems I-110-200), (R)-MG132 (Sigma M8699). Experiments were performed in biological triplicates with n = 3 wells per treatment, n = 9 in total, unless otherwise specified.

### RNA preparation and sequencing

Cell lysis and RNA purification was performed using RNeasy micro kit (Qiagen) following the kit manual and supplementing the cell lysis buffer with beta-mercaptoethanol. RNA quality control, library generation and mRNA NGS sequencing was performed by Exiqon Services A/S, Denmark. RNA quality control was performed using RNA ScreenTape (Agilent Technologies). Library preparation was done using TruSeq® Stranded mRNA Sample preparation kit (Illumina Inc). mRNA was enriched using oligodT bead system. First strand synthesis and second strand synthesis were performed and the double stranded cDNA was purified (AMPure XP, Beckman Coulter). The cDNA was end repaired, 3′ adenylated and Illumina sequencing adaptors ligated onto the fragments ends, and the library was purified (AMPure XP). The mRNA stranded libraries were pre-amplified with PCR and purified (AMPure XP). The libraries size distribution was validated and quality inspected on a Bioanalyzer high sensitivity DNA chip (Agilent Technologies). High quality libraries were quantified using qPCR, the concentration normalized and the samples pooled. The library pools were re-quantified with qPCR and optimal concentration of the library pool used to generate the clusters on the surface of a flowcell before sequencing using Nextseq500 High Output sequencing kit (Illumina Inc.) with 50 nucleotide, single-end read with a sequencing depth of 25 million reads. Samples with 28 s/18 s rRNA ratios below two and samples with RIN below five were excluded from analysis. Sequences were mapped in CLC Gemonics Workbench 9.0.1 (Qiagen Denmark) against *mus musculus* GRCm38 genome. Data analysis was performed using CLC Genomics Workbench 10.0.1 and CLC Main Workbench 7.9.1 (Qiagen A/S). A gene expression TPM cut-off value was set at the 25% percentile categorising genes with expression below this limit as noise. Genes with expression above the 25%, the median or the 75% percentile were defined as having low, medium or high expression, respectively. Treatments were performed as described in tau internalisation and degradation experiments. Samples were collected from four biological replicates with the following final number of samples included in analysis: C10.2 + P3 (n = 5), IgG1 control + P3 (n = 4), P3 + PBS (n = 3), C10.2_D265A_ + P3 (n = 3), untreated (n = 3), LPS 100 ng/ml (n = 4), LPS 10 µg/ml (n = 5).

### Flow cytometry analysis

Microglia used for flow cytometry were collected directly after orbital shaking and transferred to eppendorf tubes stored on ice. Cells were stained using APC-Cy7 LIVE/DEAD Fixable Dead Cell kit (ThermoFisher). ArC Amine Reactive Compensation Bead Kit (ThermoFisher) and BD CompBeads (BD Biosciences) were used for calibration of the flow cytometer. Cells were stained using Alexa Fluor 488 conjugated Rat Anti-CD11b (BD Biosciences) and isotype control and fixed using 4% PFA. Flow cytometry was performed on FACSverse (BD Biosciences) gating for single cells, identified by forward and side scatter profile followed by exclusion of dead cells. 10,000 live cells were recorded.

### Statistics

All RNA-Seq data (all mRNA data) is reported as mean with error bars showing the entire data range. All statistical comparisons of RNA-Seq data were made in CLC Genomics Workbench version 10 using proportion-based statistical comparison using Baggerley’s test, and all reported p-values are false discovery-rate-corrected (FDR) p-values, unless otherwise specified. All other statistical comparisons of tau and antibody numbers were performed using GraphPad Prism version 4.02 using one-way ANOVA, with Tukey’s posttest, unless otherwise specified. One, two or three stars represent *p* < *0.05*, *p* < *0*.*01* and *p* < *0.001*, respectively.

## Results

### Characterisation of primary microglial cultures

**Figure 1:**
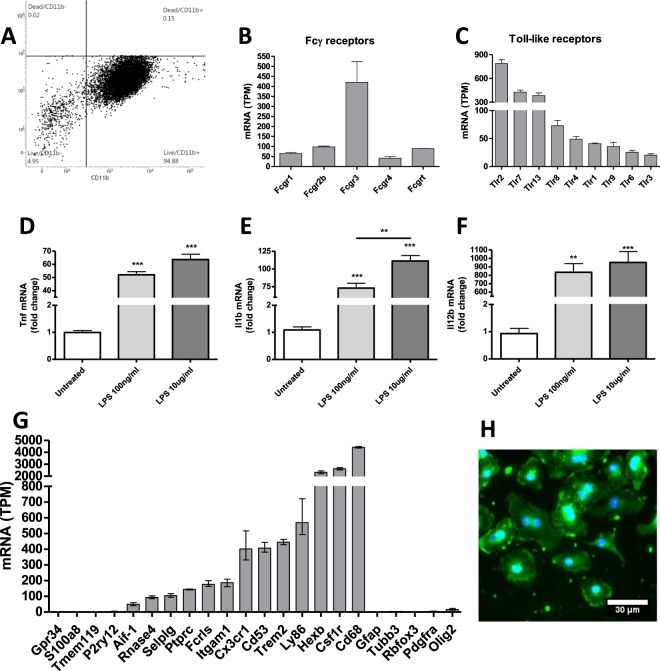
Characterization of primary microglia culture from P1 mice. (**A**) Flow-cytometry performed on cultured microglia showed that 94.88% of live cells (APC-Cy7^low^) were CD11b positive (CD11b^+^). Gene expression profiling using RNA-Seq revealed that the cells expressed mRNA for (**B**) all Fcγ receptors and (**C**) Toll-like receptors (TLR). Following challenge with lipopolysaccharide (LPS) for 3 hours cells up-regulated mRNA encoding the proinflammatory cytokines (**D**) *Tnf*, (**E**) *Il1b* and (**F**) *Il12b* in an LPS concentration-dependent manner. (**G**) Gene expression profiling using RNA-Seq revealed low to medium expression of markers of immature and mature neurons and oligodendrocytes *Tubb3* (βIII Tubulin), *Rbfox3* (NeuN), *Pdgfra* (Platelet Derived Growth Factor Receptor Alpha) and *Olig2* (Oligodendrocyte Transcription Factor 2). High expression of several microglia mRNA markers was observed and low expression of mature microglia markers Gpr34, S100a8, Tmem119, P2ry12. (**H**) Immunocytochemistry verifying cellular expression of Iba-1 colocalizing with nuclei. Bars in (**B**,**C**,**G**) are mean values plotted with entire range of dataset. Bars in (**D–F**) are mean (SEM). Statistics was performed using one-way ANOVA with Tukey**’**s posttest **p < 0.*01* ***p < *0.001*. Data in (**A**,**H)** were produced in one biological replicate each.

Primary culture characterisation was performed by flow-cytometry, immunocytochemistry, cytokine mRNA profiling following LPS challenge and by gene expression profiling using RNA-Seq. Characterisation of the cultures showed that cells stained positive for Iba-1 and that 94.8% of viable cells expressed CD11b (Fig. 1A,H). We further performed gene expression profiling of the culture using RNA-Seq, categorizing gene expression as low, medium or high defined as mRNA expression above the 25%, 50% or 75% percentile of TPM values for the entire dataset, respectively. Gene expression showed high mRNA expression for all four murine Fcγ receptors and FcRn as well as mRNA for several Toll-like receptors (Fig. 1B,C). Upon challenge with lipopolysaccharide (LPS) microglia up-regulated transcription of genes encoding pro-inflammatory cytokines *Tnf*, *Il1β* and *Il1*2*β* in an LPS concentration-dependent manner (Fig. 1D–F), as would be expected from LPS-activated microglia. RNA-Seq revealed high mRNA expression of several microglial markers (Fig. 1G) amongst others *Aif-1* (Iba-1), *Ptprc* (CD45), *Itgam1* (CD11b), *Cx3cr1*, *Cd68, Trem2* while showing low to medium expression of markers of potentially contaminating immature and mature oligodendrocytes *Pdgfra* and *Olig2*, respectively.

Low expression values was observed for markers of immature and mature neurons *Tubb3* (βIII Tubulin) and *Rbfox3* (NeuN), respectively. Expression of the astrocyte marker *Gfap* did not exceed the 25% percentile limit defined as the TPM cut-off level for noise. Expression of the microglia markers *Gpr34*, *S100a8*, *Tmem119* and *P2ry12* was low. This reflects that microglia obtained from one day postnatal mouse pups are less mature and do not show a gene expression profile similar to mature microglia extracted from aged mice when cultured *in vitro*^33^. For instance gene expression levels of the two mature microglia markers *Tmem119* and *P2ry12* have been shown to rapidly down-regulate when microglia are cultured *in vitro*^34^, and *Tmem19* is only expressed after three to six days postnatal^35^. Together these data show that the culture recapitulates the gene expression profile expected from *in vitro* cultured primary microglia and show a functional response to LPS treatment that would be expected from these cells, while showing modest gene expression for markers of potentially contaminating cells in the culture.

### Intracellular antibody-mediated degradation of P3 tau in microglia

**Figure 2:**
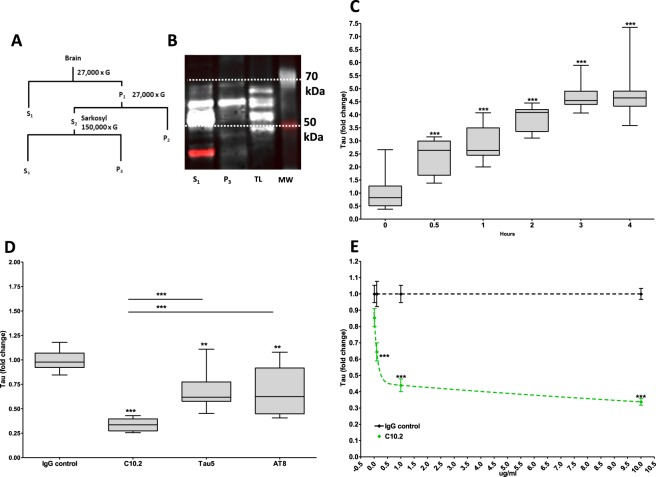
Anti-tau antibodies lower P3 tau levels in microglia in a concentration-dependent manner. (**A**) Representation of P3 tau centrifugation protocol used to obtain sarkosyl-insoluble hyper-phosphorylated P3 tau [adapted from^29^]. S and P represents supernatant and pellet, respectively, and the number in subscript represent the number of centrifugations steps the sample has been through. (**B**) Representative western blot showing that isolated P3 tau fractions (P3) were enriched for the 64 kDa tau band characteristic for hyper-phosphorylated tau when compared to S1 fractions (S1), TL and MW is recombinant tau ladder and molecular weight marker, respectively. (**C**) Microglia incubated with P3 tau accumulate tau in a time-dependent manner. (**D**) 10 µg/ml of C10.2 significantly lowers tau levels in microglia when compared to IgG control and the two commercially available total- and phospho-tau antibodies Tau5 and AT8, respectively. (**E**) C10.2 lowers tau in microglia in a concentration-dependent manner with concentrations ranging from 0.01 to 10.0 µg/ml when compared to IgG control. Error bars are mean (SEM). C10.2-mediated tau degradation was fitted using a two-phase exponential decay equation (R^2^ = 0.7084). Data in (**C**–**E**) are pooled data from three biological replicates with a total n = 9 for each treatment. Prior to pooling data were normalized to the mean value of the IgG control group in the respective experiments. In (**C**) data were further normalized to time point 0 hours. Statistics: in (**C**) repeated measures one-way ANOVA, in (**D**) one-way ANOVA, in (**E**) two-way ANOVA with Bonferroni posttest. Tukey**’**s posttest was used for group comparisons in (**C**,**D**). Two or three stars represent *p* < 0.01 and *p* < 0.001, respectively. In (**C**) statistical comparison is made between 0 hour time point and remaining time points. In (**D**,**E**) statistical comparisons are made between the specified antibody and the IgG control antibody in the same concentration or intra-group comparisons indicated by horizontal lines.

Sarkosyl-insoluble hyperphosphorylated tau fractions (P3) were isolated by stepwise ultracentrifugation of brains from 10-month-old rTg4510 mice over-expressing human 0N4R P301L tau (Fig. 2A). The isolated fractions were enriched for the 64 kDa phospho-tau band (Fig. 2B) associated with hyper-phosphorylated tau^29^. By using a Cellomics Arrayscan, an automated high-throughput immunocytochemistry quantification setup, tau uptake was measured in microglia. When incubated with P3 tau, microglia were able to take up and accumulate tau in a time-dependent manner resulting in a significant 4.8 ± 1.03 (SD) fold increase (*p* < 0.001) after four hours when compared to the first time point (Fig. 2C, Supplementary Fig. 3). To investigate if antibodies targeting pathological tau facilitates IAMD of tau in primary microglia cultured *in vitro*, cells were treated with P3 tau in complex with the phospho-serine-396-specific (pS396) anti-tau IgG1 isotype antibody C10.2. Addition of C10.2 resulted in a 66.2% reduction in tau staining when compared to microglia receiving tau in combination with an IgG control (*p* < 0.001). Tau IAMD could be reproduced using two commercially available antibodies targeting total- and phospho-tau, Tau5 and AT8 respectively. Addition of Tau5 and AT8 resulted in a 30.6 and 32.1% reduction in staining (*p* < 0.01), respectively, that was significantly higher than the levels obtained with C10.2 (*p* < 0.001) (Fig. 2D, Supplementary Fig. 4). Using 0.01 to 10 µg/ml of C10.2, it was established that tau clearance could be induced in an antibody concentration-dependent manner when comparing to IgG control (Fig. 2E).

### Antibody-dependent lowering of tau requires FcγR binding

**Figure 3:**
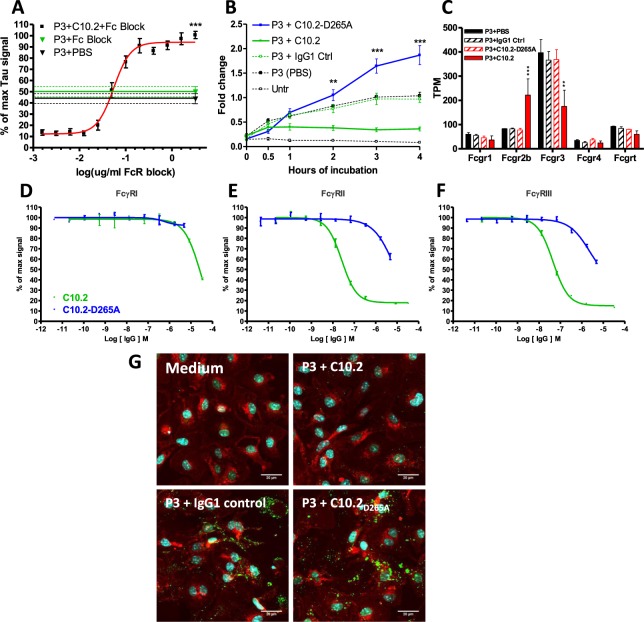
Antibody-dependent lowering of tau requires FcγR interaction. (**A**) Competitive inhibition of C10.2-mediated tau clearance by blocking FcγRII/III using anti CD16/32 antibody. Microglia were incubated with P3 tau and 10 µg/ml of C10.2 in combination with concentrations of Fc Block ranging from 1.56 ng/ml to 3.2 µg/ml. All concentrations of Fc Block above 25 ng/ml (data from middle of curve and up) resulted in a highly significant (*p* < 0.001) reversal of C10.2-mediated tau lowering when compared to the lowest concentration of Fc Block (stars are not depicted in graph). Maximum reversal of C10.2-mediated clearance was observed with 3.2 µg/ml of Fc Block at which a highly significant (*p* < 0.001) ~2-fold increase of tau levels could be observed when compared to P3 in combination with antibody solvent (PBS) or 3.2 µg/ml Fc Block. Values are mean (SEM). (**B**) Incubation of microglia with P3 tau results in a time-dependent accumulation of tau that can be prevented by 10 µg/ml of C10.2, but not IgG control. C10.2-mediated tau lowering is dependent on FcγR interaction as the non-FcγR binding antibody C10.2_D265A_ significantly reverses antibody-mediated clearance when compared to C10.2 and results in a 1.9-fold increase in tau levels when compared to IgG control. Statistics: two-way repeated measures ANOVA with Bonferroni posttest. (**C**) Microglia treated with P3:C10.2 immune complexes upregulate *Fcgr2b* (*p* < 0.001) and down-regulate *Fcgr3* (*p* < 0.01) in a manner that is dependent on antibody interaction with FcγRs as C10.2_D265A_ does not change expression levels. (**D**–**F**) Affinity measurement of monomeric C10.2 and C10.2_D265A_ for (**D**) FcγRI, (**E**) FcγRIIb or (**F**) FcγRIII. C10.2 data followed a sigmoidal dose-response curve (green) showing affinity for FcγRIIb/III, but low affinity for FcγRI. C10.2_D265A_ (blue) showed reduced affinity for FcγRIIb/III. Values are mean(SD). (**G**) Treatment with C10.2 lower tau levels when compared to IgG control, in a manner that is dependent on binding of FcγRs as treatment with C10.2_D265A_ does not lower tau. Immunocytochemistry coloring; red: WGA, green: tau, cyan: nuclei.

As microglia are phagocytosing cells expressing all four Fcγ-receptors both at protein^22^ and mRNA levels (Fig. 1B), we hypothesised that the antibody-mediated lowering of tau required binding to FcγRs. In order to test this hypothesis, we tested an effectorless version of C10.2 (C10.2_D265A_) containing the variable domains of C10.2 in combination with an Fc domain with compromised FcγR binding. Treatment with C10.2_D265A_ abolished the tau-lowering effect previously observed (Fig. 3B,G). Interestingly, addition of C10.2_D265A_ not only prevented the antibody-mediated lowering of tau and reversed it to IgG control levels, but also appeared to increase the tau levels ~2 fold over tau in combination with IgG control (Fig. 3B). To test if the increase in tau levels was an intrinsic property of the C10.2_D265A_ molecule, we further validated this finding by competitive binding using the monoclonal anti-mouse CD16/CD32 antibody Fc Block (BD Pharmingen) targeting low-affinity FcγRs CD16 (FcγRIII) and CD32 (FcγRIIb). Abolishing FcγR interaction by competitive binding resulted in a concentration-dependent loss of the previously observed tau reduction in microglia, demonstrating that binding to low-affinity FcγRIIb and/or III is required to elicit antibody-mediated tau clearance in the assay (Fig. 3A). As previously observed when using C10.2_D265A_, preventing FcγR binding not only abolished the antibody-dependent tau-lowering effect of C10.2 but also resulted in ~2 fold (*p* < 0.001) higher amounts of tau levels in the cells than controls treated with P3 tau in combination with either 3.2 µg/ml Fc Block or PBS (Fig. 3A). As antibody immune complexes have been shown to up-regulate FcγRIIb and III *in vivo* following anti-Aβ antibody treatment^36^, we tested if the transcriptional levels of these receptors were also changed *in vitro* using anti-tau antibodies in complex with pathological P3 tau. Using RNA-Seq we observed a significant 2.6 fold up-regulation of mRNA encoding *Fcgr2b* (*p* < 0.001) and a 2.1 fold down-regulation of *Fcgr3* (*p* < 0.01) following treatment with C10.2 immune complexes when comparing to P3 tau in combination with IgG1 control or PBS. The observed change in FcγR expression was absent when treating cells with C10.2_D265A_ (*p* < 0.01) showing that antibody binding to FcγRs drives the observed transcriptional regulation of these FcγRs (Fig. 3C). We determined the affinity profile of monomeric C10.2, which is an IgG1 antibody, for mouse FcγRI, IIb, and III using a FRET-based competition assay. In line with the data obtained using Fc Block, C10.2 had a binding profile favouring interaction with low-affinity FcγRIIb (EC_50_: 2.59·10^−8^ M) and III (4.51·10^−8^ M) over FcγRI (2.86·10^−5^ M) (Fig. 3D–F). By testing C10.2_D265A_ in the assay we also confirmed that C10.2_D265A_ had compromised binding for FcγRIIb and III (Fig. 3D–F).

### Lysosomal function is required for tau clearance

**Figure 4:**
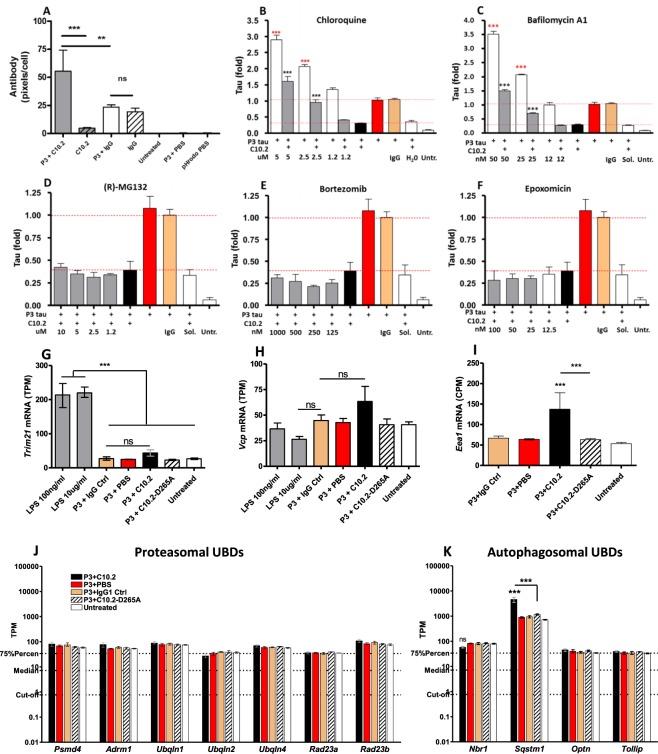
Tau degradation can be prevented by pharmacological inhibition of lysosomes. Using automated high-throughput quantification of tau immunocytochemistry and pHrodo staining, it was shown that. (**A**) C10.2 antibodies are internalised into acidic compartments. Both unbound (striped) and immune complex-bound C10.2 (solid bars) was taken up. No signal was obtained for untreated cells, cells receiving P3 tau and antibody solvent (P3 + PBS) or cells treated with a labeling control of PBS (pHrodo PBS) controlling for un-conjugated pHrodo in solvent. Lysosomal inhibitors (**B**) chloroquine and (**C**) bafilomycin A1 resulted in a concentration-dependent increase in tau levels in antibody-treated and non-antibody-treated microglia. (**D–F**) Inhibition of proteasomes did not prevent IAMD. Numbers under graphs represent inhibitor concentration. *IgG*: control IgG, H_2_O: *H*_*2*_*O* solvent, *Sol*.: DMSO solvent, *Untr*.: untreated cells. (**G**) *Trim21* mRNA levels are up-regulated following LPS challenge, but not by C10.2 treatment. (**H**) *Vcp* mRNA levels are not affected by antibody or LPS treatment. (**I**) *Eea1* mRNA is significantly increased following C10.2 treatment but not by C10.2_D265A_. Transcriptional levels of mRNA encoding Ubiquitin-Binding Domain (UBD) adaptor proteins linking ubiquitin-bound substrates for (**J**) proteasomal and (**K**) autophagosomal degradation. No increase observed in UBDs involved in proteasomal degradation, but one gene involved in autophagosomal degradation (*Sqstm1*) was significantly increased in an FcγR-dependent manner. Concentration of antibodies: 10 µg/ml in all graphs. Red horizontal lines indicate IAMD window. (**B**,**C**) represent pooled data from three biological replicates (total n = 9 wells per treatment), bars are mean(SEM). In (**B**,**C**) black stars are comparison of values obtained from cells treated with C10.2 in combination with inhibitors (gray bars) versus C10.2 without inhibitors (black bars). Red stars indicate comparison of cells receiving P3 tau plus inhibitors (white bars) versus P3 tau only (red bars). Data in (**A**,**D–F**) was made in one experimental replicate (n = 3 wells per treatment). Bars are mean(SD). Statistics: one-way ANOVA with Tukey**’**s posttest for relevant comparisons. Data in (**G**–**I**) are mean value with range.

Phagocytosis of antibody-coated and non-antibody coated antigens by microglia results in trafficking through the endolysosomal pathway^23^. We wanted to verify if this was also the case for C10.2 bound to tau. Using the Cellomics Arrayscan setup, we quantified fluorescently labelled antibodies in microglia using the pH-sensitive phagocytosis marker pHrodo which emits light in a pH-dependent manner. Using this approach, we verified that C10.2 was internalised into a low-pH compartment (Fig. 4A). We also showed that immune complexes resulted in transcriptional up-regulation of mRNA encoding the marker of early endosomes (*Eea 1*) in a manner that depended on FcR interaction indicating uptake through the endolysosomal pathway (Fig. 4I). As lysosomal proteolytic cleavage of internalised proteins is dependent on acidification of the vesicles to create a pH optimum for lysosomal proteases, we tested if pharmacological inhibition of lysosome acidification prevented antibody-mediated degradation of internalised tau. Using different concentrations of chloroquine and bafilomycin A1 we were able to abolish the degradation of tau in a concentration-dependent manner. Interestingly, at the highest concentrations of inhibitors, the clearance was not only reversed to IgG control levels, but also exceeded the treatment window as it was increased to ~1.5 fold for both inhibitors when compared to the IgG control (Fig. 4B,C). We compared the results with data obtained from cells treated with P3 tau and inhibitors in the absence of C10.2. Treatment of cells with P3 tau and antibody solvent (PBS) in combination with either of the inhibitors resulted in a concentration-dependent increase in tau levels ranging from 2.9 to 3.5 fold for the highest concentration of inhibitors when compared to the P3 tau control (Fig. 4B,C). As tau in our setup is readily accessible from the medium, these data suggest that microglia have a baseline turnover of tau that can be inhibited by targeting the lysosome resulting in accumulation of tau in the cell. It was not possible to distinguish between antibody-dependent and antibody independent degradation in the experiment. Using the Cellomics Arrayscan setup, we quantified tau immunocytochemistry in microglia treated with various concentrations of the three proteasomal inhibitors (R)-MG132, bortezomib and epoxomicin. Doing so, we tested if inhibition of the proteasome abolished tau clearance, which was not the case (Fig. 4D–F). To validate the findings and to indicate if ubiquitin-mediated degradation could be involved in antibody-mediated tau clearance, we investigated the mRNA levels of Ubiquitin-Binding Domain (UBD) adaptor proteins known to link ubiquitin-bound substrates to proteasomal and autophagosomal degradation^37^. No transcriptional change was observed for genes encoding UBD adapors destining ubiquitin-bound substrates for proteasomal degradation (Fig. 4J). However, when investigating transcriptional levels of genes encoding UBD adaptors destining ubiquitin-bound substrates for autophagosomal degradation, p62 (*Sqstm1*) was significantly up-regulated following C10.2 treatment in a manner that was dependent on interaction with Fc-receptors (Fig. 4K).

**Figure 5:**
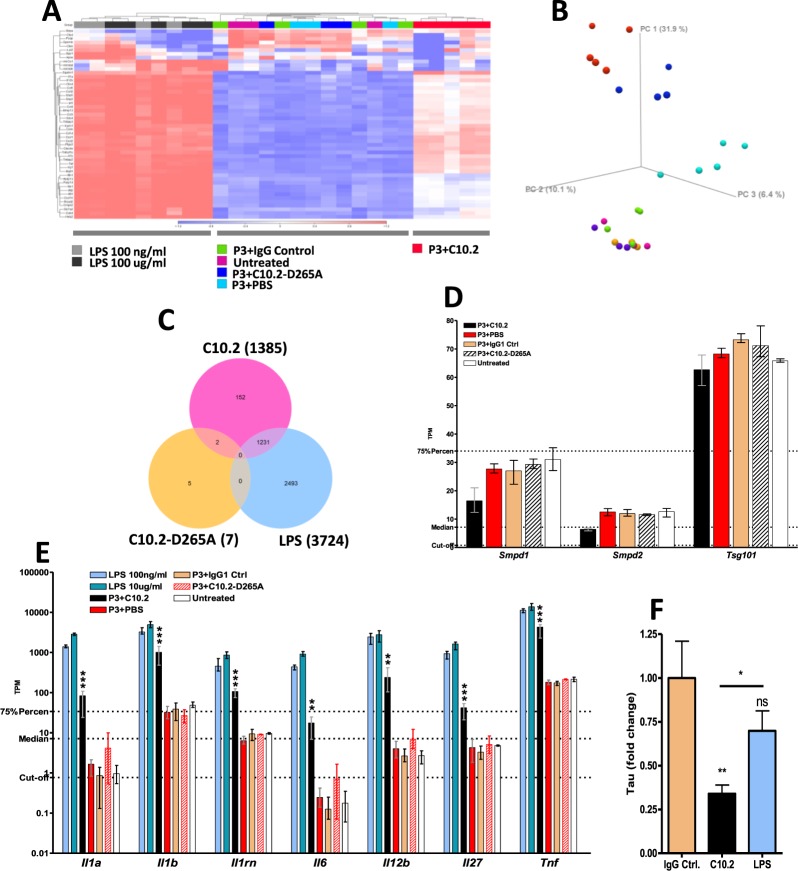
Microglial activation markers and tau degradation following C10.2 and LPS treatment. (**A**) C10.2, LPS or control-treatments of microglia result in gene expression profiles that cluster different on heat map and (**B**) separate into three separate clusters on principle component analysis plot. Samples treated with low and high LPS concentrations (blue and red, respectively) cluster in the top of the plot. Samples treated with C10.2 immune complexes (light blue) cluster in the right side of the plot. All other treatments cluster in the bottom. (**C**) Venn-diagram showing the number of genes differentially expressed by microglia treated with P3 tau in complex with either C10.2 or C10.2_D265A_ (C10.2-D265A) versus P3 + IgG control or LPS alone versus P3 + IgG control. Only genes with *FDR p-value* < *0.05* and a minimum absolute fold change of 2 were included. (**D**) mRNA expression values of genes involved in exosome biogenesis were not significantly up-regulated following C10.2-treatment. C10.2 lowered mRNA expression, but the effect was not significant. (**E**) Treatment of microglia with P3 + C10.2 induced significant up-regulation of pro-inflammatory markers which was dependent on FcγR interaction as C10.2_D265A_ did not induce up-regulation. Statistical comparison is between C10.2 and C10.2_D265A_. LPS was included as a positive control to identify the maximum level of activation of microglia as assessed by mRNA encoding pro-inflammatory cytokines. Both concentrations of LPS resulted in significant (*p* < *1·10*^−5^) up-regulation of all genes (stars are not shown on graph). *Il-6* was not included in LPS comparison as the mean TPM of the untreated control was below the defined expression cut-off. (**F**) Treatment of microglia with P3 tau in complex with C10.2 or 10 µg/ml IgG control or P3 in combination with 100 ng/ml LPS. C10.2, but not LPS, induced a significant clearance of tau in microglia in our assay. Experiment in (**F**) was performed in one replicate. *p < *0.05*, ** *p* < 0.01, ***p < *0.001*.

The cytoplasmic Fc receptor TRIM21 has previously been shown to act as a detector of cytoplasmic antibodies and facilitate proteasomal clearance of antibody-coated pathogens present in the cell cytoplasm in a manner that is dependent on the valosin-containing protein (VCP)^38,39^. As TRIM21 mRNA and protein amounts are closely correlated^40^, we tested if treatment with P3 tau:C10.2 immune complexes induced significant up-regulation of *Trim21*, indicative of cytoplasmic presence of antibodies. We furthermore tested if treatment induced up-regulation of mRNA encoding *VCP*, but none of these mRNAs were significantly up-regulated by the treatment (Fig. 4G,H). Primary microglia have previously been reported to spread tau through exosomes *in vitro* when using a M-CSF-based culturing approach comparable to ours^41^. We investigated the transcriptional levels of genes involved in exosome biogenesis^23,41,42^ to test if C10.2-mediated tau reduction was accompanied by an increase in mRNA markers of exosome formation, indicative of spreading rather than breakdown. C10.2-treatment did not increase the expression levels of genes in the ESCRT (*Tsg101*) or sphingomyelinase (*Smpd1/2*) pathways (Fig. 5D).

### Antibody-mediated clearance of tau activates microglia in an Fc-dependent manner

Using RNA-Seq we investigated gene expression changes in microglia following treatment with C10.2 immune complexes. Treatment with C10.2 immune complexes resulted in a gene experssion profile that clustered differently on a heatmap than controls or LPS-treated microglia and separated into distinct clusters on a PCA plot (Fig. 5A,B). Cells treated with C10.2 or LPS showed a significant up-regulation (*p-value* < *0.01*) of mRNA encoding pro-inflammatory cytokines such as TNF, IL-1β and IL-12b. This increase in expression was not observed for cells treated with C10.2_D265A_ (Fig. 5E). To investigate if activation of microglia was sufficient to reduce tau levels to the same extent as in antibody-treated cells, we compared tau levels in cells treated with P3 tau in combination with LPS, C10.2 or IgG control. In contrast to C10.2, LPS did not significantly reduce tau levels in the cells (Fig. 5F). To identify genes involved in antibody-mediated tau clearance, we determined the overlap of gene expression profiles of LPS-activated cells and cells treated with C10.2. We found that LPS and C10.2 treatment resulted in 3724 and 1385 differentially expressed genes, respectively. 1231 of genes up-regulated by C10.2 immune complexes overlapped with LPS-up-regulated genes and 152 genes were exclusively upregulated by C10.2 (Fig. 5C).

## Discussion

Antibody-mediated prevention or reduction of tau pathology in tauopathy models has been the focus of many studies^14–17,43^. Anti-tau antibodies have been suggested to act through different mechanisms: prevention of aggregation of tau, prevention of cell-to-cell spreading or by induction of cellular degradation of tau. Microglia are the primary phagocytic cells in the brain, thus understanding microglial antibody-mediated clearance is essential to harness the therapeutic potential of these cells. Previous studies conducted using anti-tau IgG2a/b isotype antibodies have shown that it is possible to increase tau uptake in microglia or BV2 cells in a manner that is dependent on FcγR interaction^20,21^. However, these studies have only adressed the accumulation of tau in microglia, or the disapperance from the surronding medium, and not if tau undergoes antibody-mediated intracellular clearance once internalised. Funk *et al*.^20^ observe an increased uptake of tau into BV2 cells when treating with anti-tau IgG2 antibodies HJ8.5 and HJ9.4. However, the tau amounts in cells is decreased when blocking low affinity FcγRs. This is in contrast to our findings as we observe the opposite effect. Similarily Lou *et al*.^21^ see a reduction of tau in medium (*i.e*. microglial uptake) in the presence of anti-tau antibody MC1, but do not see a reduction of tau from the medium when using MC1 Fab fragments. Together these studies show that microglial uptake of tau can be facilitated by anti-tau antibodies, but do not adress intracellular antibody-mediated degradation. Using mouse primary microglia cultured *in vitro*, we show by quantitative means that microglia take up and accumulate pathological tau in a time-dependent manner. We further show that by treating cells with tau in complex with IgG1 isotype antibodies targeting total- and phospho-tau, it is possible to induce clearance. Using C10.2 which specifically targets pathological hyper-phosphorylated tau^43^, we show that it is possible to obtain superior clearance when comparing to commercial antibodies, in a manner which is dependent on antibody concentration and interaction with low-affinity FcγRs. We show that blocking or preventing binding to FcγRs results in accumulation of tau in microglia demonstrating that antibody-bound tau is internalised, and that FcγR binding is necessary to induce antibody-mediated clearance of tau. Using pH-sensitive labeling of antibodies we show that antibodies are internalised into acidic cellular compartments and demonstrate that tau clearance is dependent on functional lysosomes, as tau clearance can be prevented by pharmcological inhibition of lysososomal acidification. Lysosomal inhibition exhibited a difference in tau-levels between cells treated with and without tau antibody complexes, with levels for antibody-treated cells being consistently lower showing that the antibody may still elicit some degree of tau-lowering although the lysosome is compromised. It has been shown that interaction of IgG-coated complexes with FcγRIIb during cellular internalization mediates the speed of acidification of the phagosome as well as the speed of degradation of the complexes^44^. As the phagosome is dependent on acidification by lysosomes, it may be that C10.2 immune complexes increases phagosome acidification while lysosomal inhibitors counteract the acidification preventing a full reversal of antibody-mediated clearance of tau. An alternative explanation may be that additional intracellular antibody-mediated degradation routes besides lysosomal degradation may exist. Testing this, we show that internalised antibodies do not appear to enter the cytosol, and that the proteasome does not contribute to clearance as pharmacological inhibition of the proteasome does not reverse antibody-mediated clearance of tau. Furthermore, no change in mRNA encoding UBDs targeting proteins for proteasomal degradation could be observed following antibody treatment. We show that microglial activation in itself is not sufficient to induce significant clearance of tau. We use RNA-Seq to investigate the gene expression profile of antibody-treated microglia. Although changes in mRNA expression levels do not guarantee changes in protein expression levels, we show that mRNA activation markers do reflect the functional responses expected from microglial cells. We identify a gene expression profile of microglia capable of clearing pathological tau in an antibody-dependent manner that does not overlap with the gene expression profile of activated microglia and it is our hope that research within these genes will allow for the induction of tau clearance in a manner that causes minimal risk of inflammation.

## Conclusions

In conclusion, we show that anti-tau antibodies are able to induce microglial clearance of pathological tau in primary mouse microglia cultured *in vitro*. Antibody-mediated clearance of tau in these cells is dependent on antibody interaction with low-affinity FcγRs and functinoal lysosomes. Using RNA-Seq, we determine that treatment with antibody-tau immune complexes results in transcription of genes encoding pro-inflammatory markers but that the gene expression profile of immune complex-treated microglial is different from the gene expression profile of LPS-activated microglia.

## Supplementary information

Supplementary Dateset File

## Data Availability

The datasets generated and/or analysed during the current study are not publicly available as the C10.2 antibodies used to generate the datasets has been developed as part of an ongoing drug discovery process at H. Lundbeck A/S, but may be available from the corresponding author on reasonable request.
