# Supplementary material for: Antibody-mediated clearance of tau in primary mouse microglial cultures requires Fcγ-receptor binding and functional lysosomes
Source: Sci Rep. 2019 Mar 15;9:4658. doi: 10.1038/s41598-019-41105-4 (PMC6420568; doi:10.1038/s41598-019-41105-4)
Supplement: Supplementary file 1 — Supplementary Dateset File [file 41598_2019_41105_MOESM1_ESM.pdf]

Supplementary Dataset File

Antibody-mediated clearance of tau in primary mouse microglial cultures requires Fcγ-receptor binding and functional lysosomes

Christian Rungsted Andersson[1,2,*], Jeppe Falsig[1], Jeffrey B. Stavenhagen[3], Søren Christensen[4], Fredrik Kartberg[4], Nina Rosenqvist[1], Bente Finsen[2], Jan Torleif Pedersen[1]

1 Department of Neurodegeneration, H. Lundbeck A/S, Copenhagen, Denmark.
2 Neurobiology Research, Institute of Molecular Medicine, University of Southern Denmark, Odense, Denmark.
3 Biology, Therachon, Basel, Switzerland.
4 Biologics, H. Lundbeck A/S, Copenhagen, Denmark.
*Corresponding author

The below figure is the full membrane view of the part of the western blot shown in figure 2B

Supplementary figure 1

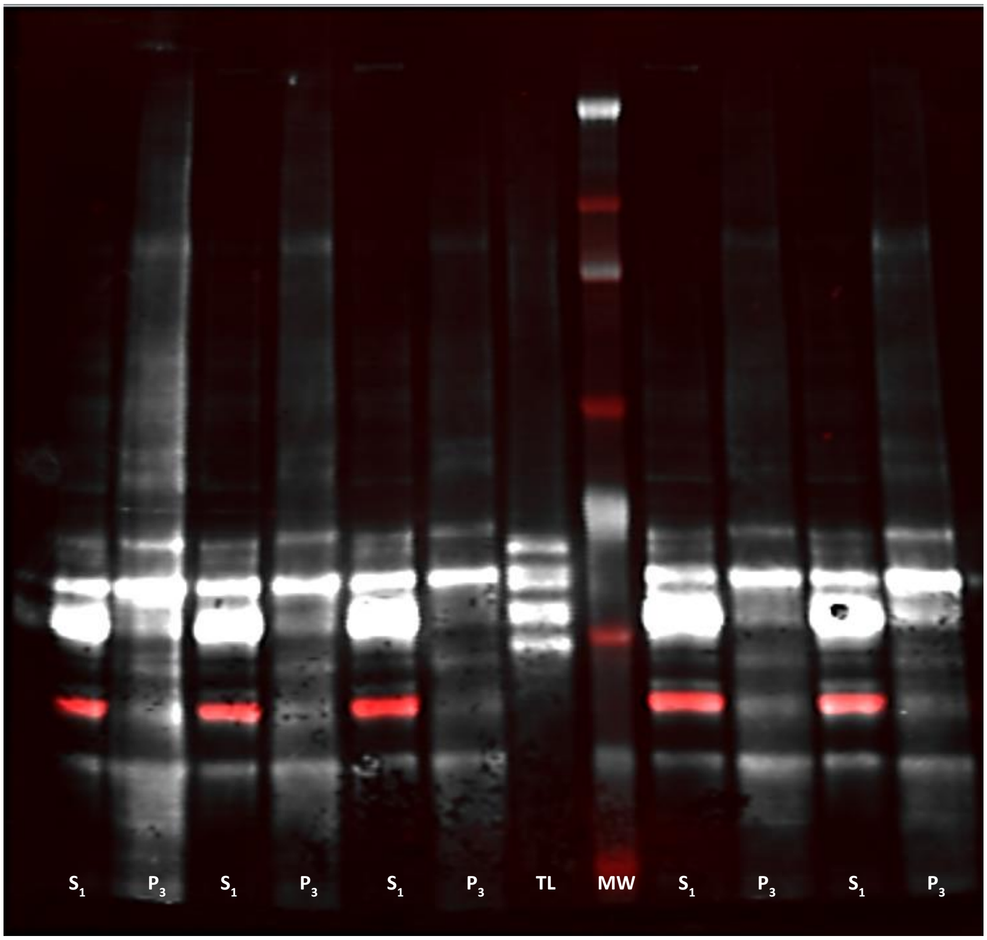

Full membrane scan of S_1 and P_3 fractions prepared from aged TG4510 mice. Adjacent S_1 and P_3 fractions are from the same animal. MW: molecular weight marker, TL: recombinant Tau ladder showing all tau 6 isoforms. White: tau, Red: actin.

Supplementary Figure 2

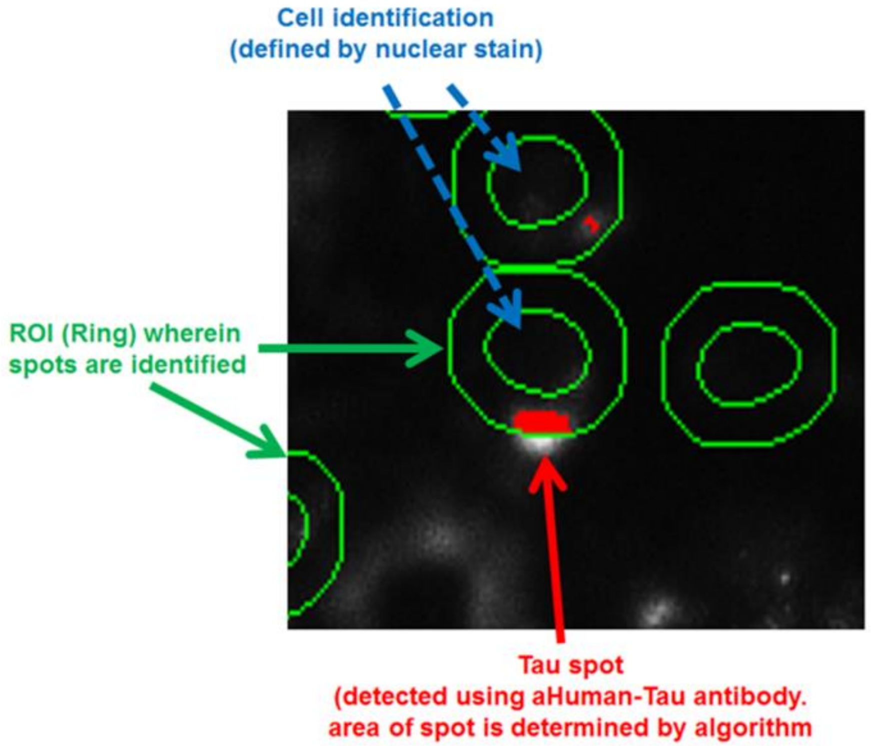

Quantification of tau immunocytochemistry using Cellomics Arrayscan VTI. By assessment of nuclear morphology and staining intensity the algorithm excludes dead cells from analysis, and quantifies the area of fluorescent spots within a defined distance from the edge of the nuclear staining. The area of tau spots with staining intensities greater than a pre-defined background level is measured. Untreated controls, and cells treated with P3 alone were used as negative and positive controls, respectively, to calibrate the algorithm. Above only tau staining is showed and the location of the nucleus and quantification ROIs are outlined by blue and green arrows, respectively. Tau staining within the ROI exceeding the pre-specified threshold is quantified (red). Tau spots outside the ROI or staining with a lower intensity are not scored.

Supplementary Figure 3

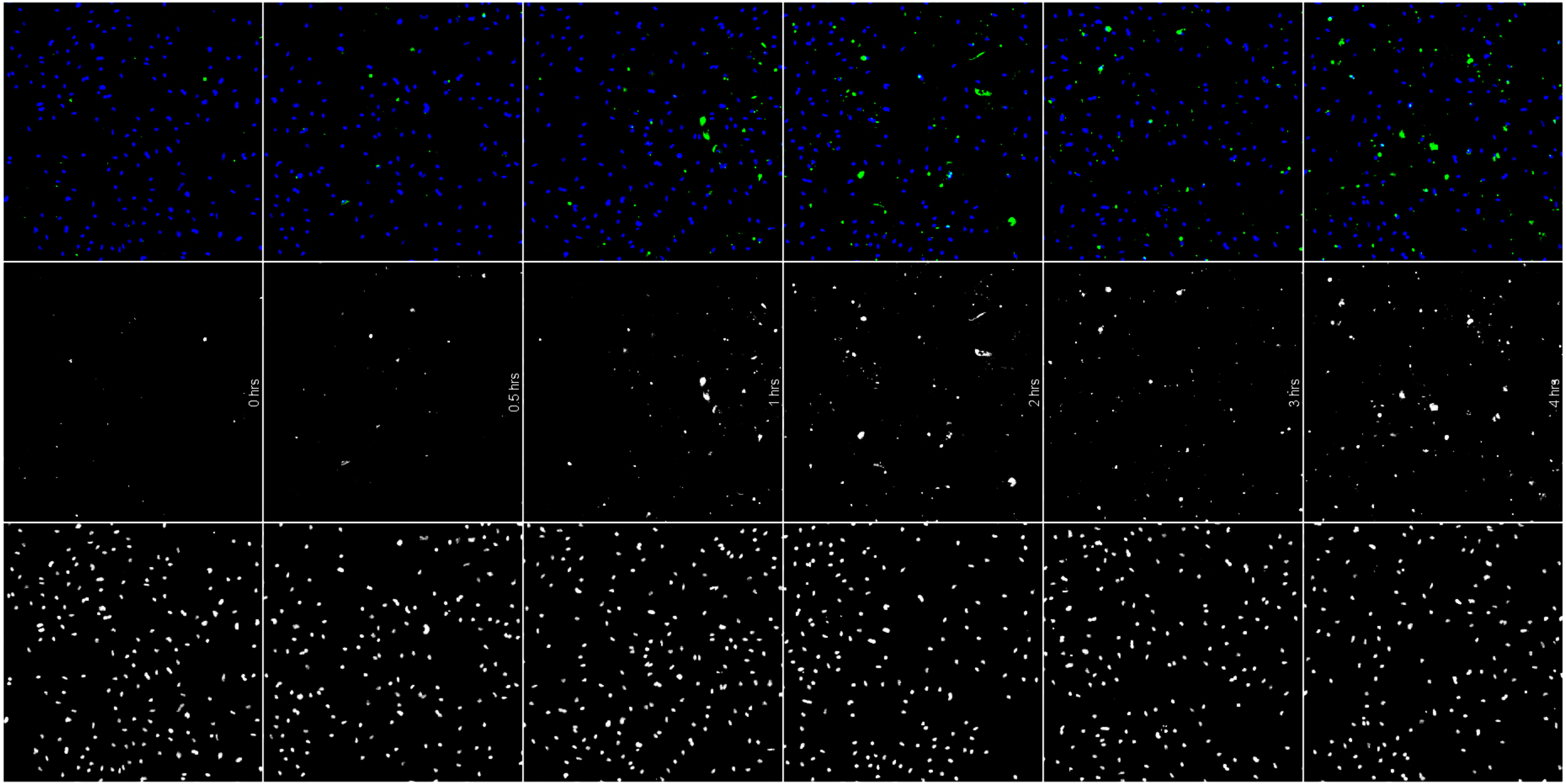

The above figure displays automatically selected images from the Cellomics Arrayscan VTI staining quantification software (the first image acquired in each well). During quantification, 16 images from each well are quantified (only one image per condition has been shown below above). The software quantifies all tau staining (pixel area) as exemplified in Supplementary Figure 2. Picture are (taken at the following time points (left to right): 0, 0.5, 1, 2, 3, and 4 hours post tau addition. Bottom row: Nuclear stain, middle row: tau stain, top row: merge. Image magnification: 20X

Supplementary Figure 4

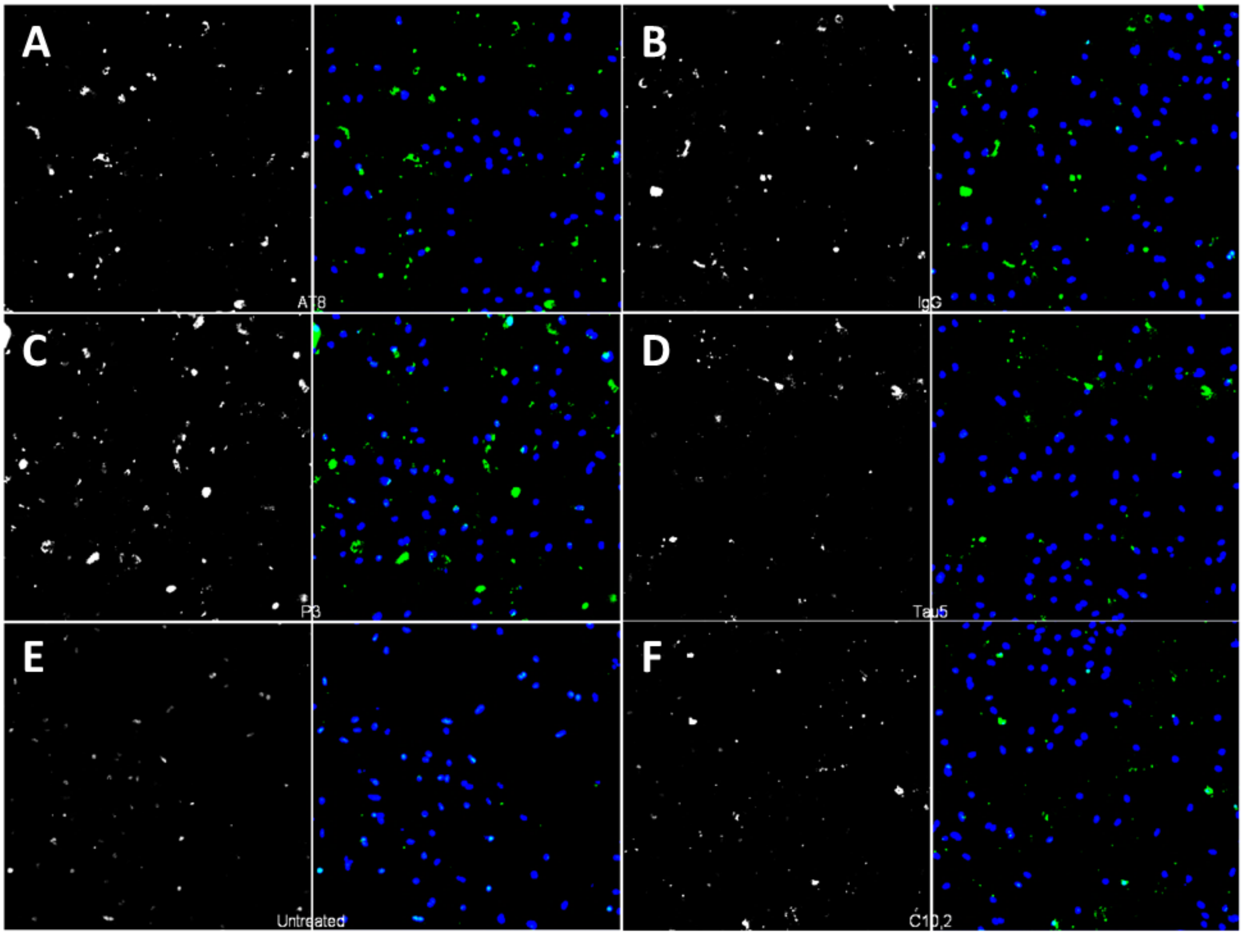

Immunocytochemistry showing some of the images quantified in Figure 2 D. The above figure displays automatically selected images from the Cellomics Arrayscan VTI staining quantification software (the first image acquired in each well). During quantification, 16 images from each well are quantified (only one image per condition has been shown below above). The software quantifies all tau staining (pixel area) as exemplified in Supplementary Figure 2. Treatments are: A) AT8, B) IgG control, C) P3 tau only, D) Tau5 , E), Untreated, F) C10.2. Pictures in black and white (left side of each treatment) shows tau staining. Merged pictures show; Tau (green), nuclei (blue). Image magnification: 20X
